# The role of circadian clock in regulating cell functions: implications for diseases

**DOI:** 10.1002/mco2.504

**Published:** 2024-03-11

**Authors:** Yanke Lin, Liangliang He, Yuting Cai, Xiaokang Wang, Shuai Wang, Feng Li

**Affiliations:** ^1^ Infectious Diseases Institute Guangzhou Eighth People's Hospital Guangzhou Medical University Guangzhou China; ^2^ Guangdong TCRCure Biopharma Technology Co., Ltd. Guangzhou China; ^3^ College of Pharmacy Jinan University Guangzhou China; ^4^ School of Pharmaceutical Sciences Guangzhou University of Chinese Medicine Guangzhou China; ^5^ Department of Pharmacy Shenzhen Longhua District Central Hospital Shenzhen China

**Keywords:** cancers, cell function, circadian clock, immune cells, inflammatory diseases, signaling pathway, systemic diseases

## Abstract

The circadian clock system orchestrates daily behavioral and physiological rhythms, facilitating adaptation to environmental and internal oscillations. Disruptions in circadian rhythms have been linked to increased susceptibility to various diseases and can exacerbate existing conditions. This review delves into the intricate regulation of diurnal gene expression and cell function by circadian clocks across diverse tissues. . Specifically, we explore the rhythmicity of gene expressions, behaviors, and functions in both immune and non‐immune cells, elucidating the regulatory effects and mechanisms imposed by circadian clocks. A detailed discussion is centered on elucidating the complex functions of circadian clocks in regulating key cellular signaling pathways. We further review the circadian regulation in diverse diseases, with a focus on inflammatory diseases, cancers, and systemic diseases. By highlighting the intimate interplay between circadian clocks and diseases, especially through clock‐controlled cell function, this review contributes to the development of novel disease intervention strategies. This enhanced understanding holds significant promise for the design of targeted therapies that can exploit the circadian regulation mechanisms for improved treatment efficacy.

## Graphical abstract

Circadian clocks regulate diurnal variations in an array of genes, delineating the roles and intracellular pathways of cells. Disruption in circadian orchestrations compromises the immune system, precipitating disturbances in cell coordination and signaling pathways, thus promoting the progression of diseases. This review indicates the rhythmicity of gene expressions, behaviors, and functions in cells and elucidates the regulatory mechanisms of cell function by the circadian clock. A detailed discussion is devoted to unraveling the multifaceted roles of circadian clocks in governing pivotal signaling pathways. We further provide a comprehensive discussion on circadian regulation across a spectrum of diseases, with a spotlight on inflammatory diseases, cancers, and systemic diseases.

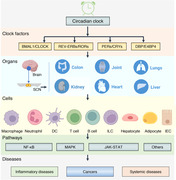

## 1 INTRODUCTION

The diurnal light/dark cycles resulting from the earth's rotation are responsible for the establishment of circadian rhythms in mammals.^1^ A wide array of behavioral phenomena, including the sleep–wake cycle, feeding behaviors, thermoregulation, cardiovascular dynamics, and hormonal secretion, exhibit a circadian rhythm with an approximately 24‐h cycle.^2^ These rhythmic patterns are orchestrated by intrinsic biological clocks. At the core of these circadian systems is a central pacemaker located in the suprachiasmatic nucleus (SCN) of the brain, complemented by subsidiary clocks in peripheral tissues.^3^ The rhythmicity is transmitted from the central to peripheral clocks via neural or hormonal signals.^4^ Intertissue communications, exemplified by the gut–brain axis, brain–heart axis, and gut–liver axis, are instrumental in maintaining circadian rhythms in gene expressions and physiological processes.^5^, ^6^, ^7^, ^8^, ^9^ For example, the intestinal clock orchestrates the hepatic rhythmic transcriptome and diurnal metabolism, thereby underpinning hepatic metabolic homeostasis.^10^ Various factors, such as brain injuries, diseases, sleep deprivation, and trans‐meridian travel result in disrupted circadian rhythm.^11^, ^12^

The circadian clock system is structured in a hierarchical manner and functions through self‐regulating feedback loops. In the main loop, the Brain and Muscle ARNT‐Like 1 (BMAL1) protein forms a complex with the circadian locomotor output cycles kaput (CLOCK) protein, creating a heterodimer.^13^ This BMAL1/CLOCK heterodimer facilitates the transcription of clock‐controlled genes (CCGs), including *Periods* (*PERs*) and *Cryptochromes* (*CRYs*), by binding to E‐box elements in their promoters.^14^ Following their synthesis, PERs and CRYs proteins accumulate and eventually exert an inhibitory effect on the BMAL1/CLOCK complex.^15^ When PERs and CRYs protein levels are reduced due to protein degradation, PERs and CRYs are dissociated from the BMAL1/CLOCK complex and a new cycle of transcription is started.^16^ Degradation of PERs and CRYs proteins is performed by casein kinases and adenosine monophosphate kinase through phosphorylation for ubiquitination and proteasome degradation.^17^ Additional feedback loops involve crucial transcriptional factors such as  reverse erythroblastosis virus heme receptors (REV‐REBs), RAR‐related orphan receptors (RORs), and D‐box acting proteins, including albumin D‐site‐binding protein (DBP) and E4 promoter‐binding protein 4 (E4BP4), which contribute to the stability and robustness of the circadian clock system.^18^, ^19^ Specifically, RORα and REV‐REBα play opposing roles and finely regulate the transcription of *Bmal1* and *E4bp4*, by binding to ROR responsive elements that are located in the promoters of these genes.^20^ REV‐ERBs repress the transcription of *Bmal1*, while RORs activate their transcription.^21^ RORs activate the expression of *E4bp4*, whereas REV‐REBs inhibit its transcription.^22^ These intricate regulatory mechanisms ensure the precise control and maintenance of the circadian clock system.

The circadian clock orchestrates a multitude of physiological functions through the modulation of cell functions and signaling pathways. This temporal governance extends to the behaviors, differentiation, and functions of immune cells, integral to both adaptive and innate immunity systems.^23^, ^24^, ^25^, ^26^, ^27^, ^28^, ^29^ Research has increasingly focused on the circadian regulation of transcriptional processes that determine gene expressions in key signaling pathways, such as nuclear factor‐κB (NF‐κB), mitogen‐activated protein kinase (MAPK), and Janus kinase/signal transducer and activator of transcription (JAK‐STAT).^30^, ^31^, ^32^, ^33^, ^34^, ^35^, ^36^ Disruptions in circadian rhythms can precipitate the malfunctioning of these regulatory pathways, potentially leading to uncontrollable inflammatory responses and diseases.^37^, ^38^, ^39^, ^40^, ^41^, ^42^

Disruption of the circadian clock enhances the susceptibility to various diseases such as inflammatory diseases, cancers, and systemic diseases.^43^, ^44^, ^45^, ^46^, ^47^, ^48^, ^49^, ^50^, ^51^, ^52^ For example, circadian oscillations significantly influence the onset and progression of various malignancies. The susceptibility to cancers induced by circadian disruption is closely associated with cell functions and behaviors.^53^, ^54^, ^55^ The temporal coordination of immune cell functions by circadian rhythms, encompassing cell functions and behaviors such as trafficking, pathogen recognition, phagocytosis, and the secretion of inflammatory cytokines, is critical in determining the diurnal variation in cancer severity.^26^, ^56^, ^57^, ^58^, ^59^, ^60^, ^61^, ^62^

In this review, we highlight the link between circadian disruptions and increased disease susceptibility, focusing on the circadian control of gene expressions and signaling pathways in various cells. The review delves into how disturbances in circadian rhythms impair cell functions and exacerbate disease progression, particularly in inflammatory diseases, cancers, and systemic diseases. It emphasizes the potential of targeted drug interventions that harness circadian regulation, offering novel insights into disease management and therapeutic development.

## 2 CIRCADIAN REGULATION OF IMMUNE CELL FUNCTION

**FIGURE 1:**
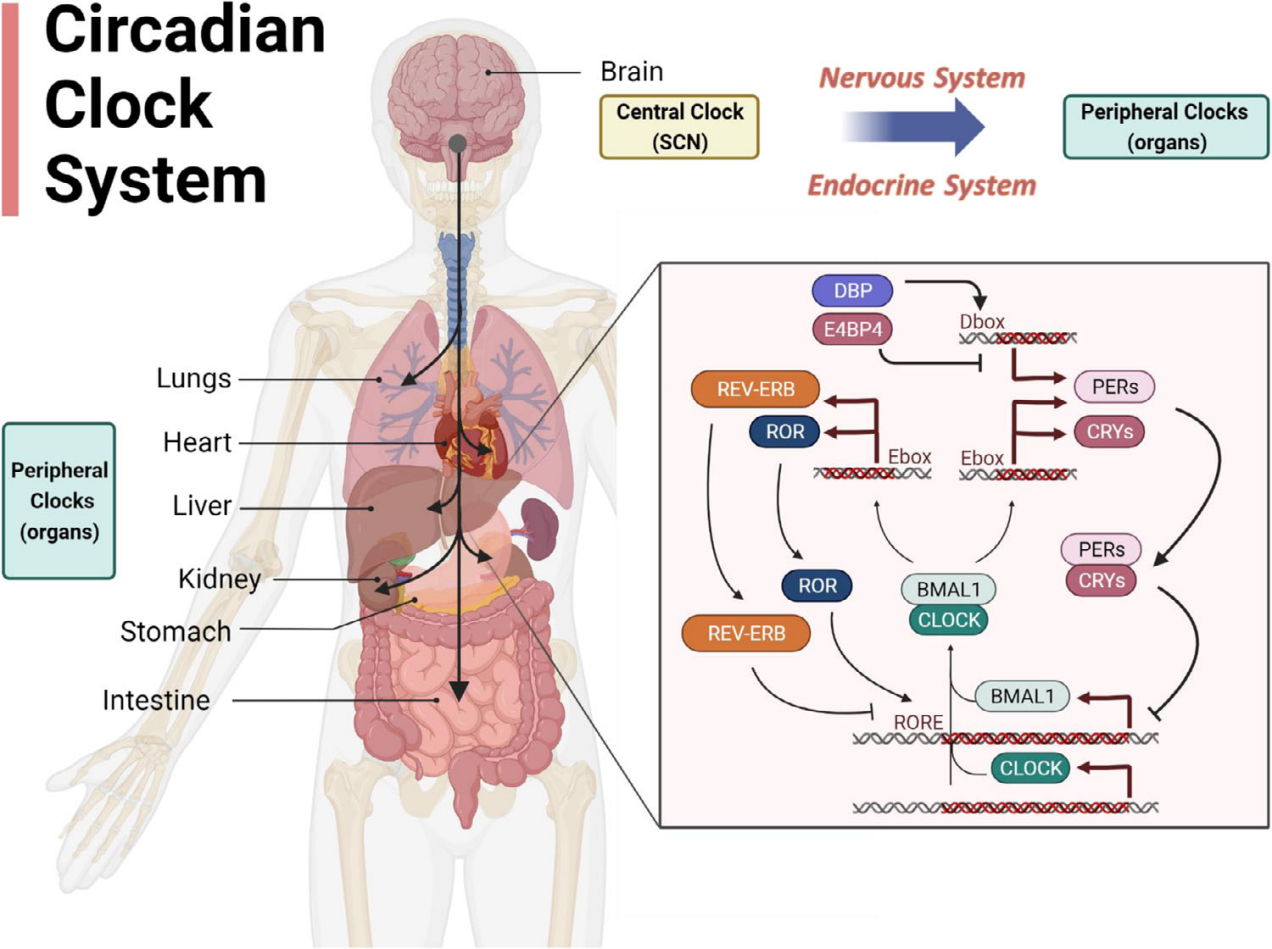
The molecular mechanism of the circadian clock system. Circadian clocks are composed of a central clock located in the SCN of the brain and peripheral clocks situated in various peripheral organs (i.e., lung, heart, liver, kidney, stomach, and intestine). Proteins encoded by clock genes *CLOCK*, *BMAL1*, *CRYs*, *PERs*, *REV‐ERB*, and *ROR* interact to create transcription‐translation feedback loops. BMAL1 interacts with CLOCK to form a heterodimer and transcriptionally regulates the expressions of CCGs (*CRYs*, *PERs*, *ROR*, and *REV‐ERB*) via E‐box. PERs and CRYs proteins accumulate and subsequently inhibit the activity of the BMAL1/CLOCK complex. RORα and REV‐REBα play opposing roles in regulating the transcription of *Bmal1* through RORE elements. DBP and E4BP4 also have inverse effects on the transcription of *PERs* through D‐box elements. BMAL1, Brain and muscle ARNT‐like 1; CCGs, clock‐controlled genes; CLOCK, circadian locomotor output cycles kaput; CRYs, Cryptochromes; DBP, albumin D‐site‐binding protein; E4BP4, E4 promoter‐binding protein 4; PERs, Periods; REV‐ERB, reverse erythroblastosis virus heme receptors; ROR, retinoic acid‐related orphan receptor; RORE, ROR‐responsive element; SCN, suprachiasmatic nucleus.

**FIGURE 2:**
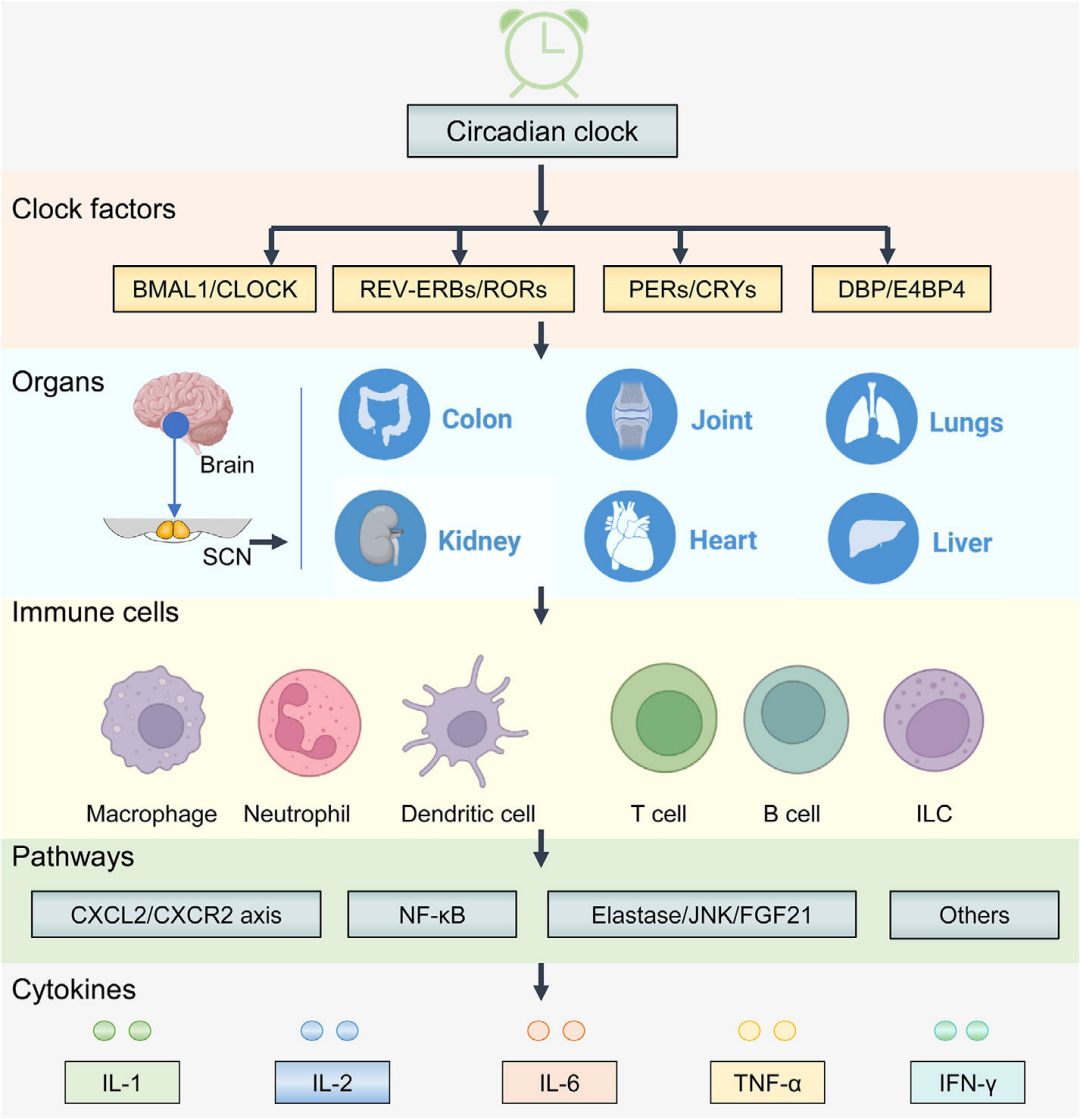
The circadian clock‐controlled immune responses. The circadian clock plays a pivotal role in the regulation of immune responses within immune cells across multiple organs. Through its influence on various signaling pathways and the release of cytokines, the circadian clock orchestrates immune responses in a diverse range of immune cell types. Within these cells, the circadian clock contributes to the precise coordination and modulation of inflammatory signaling pathways and the release of cytokines. CXCL, chemokine ligand; CXCR, CXC‐chemokine receptor; FGF21, fibroblast growth factor 21; IFN, interferon; IL, interleukin; ILC, innate lymphoid cell; JNK, c‐Jun N terminal kinase; NF‐κB, nuclear factor‐κB; TNF, tumor necrosis factor.

The biological clocks located in SCN and peripheral tissues (i.e., liver, heart, lung, kidney, stomach, and intestine) consist of several positive and negative molecular feedback loops (Figure 1). The rhythmicity is transmitted from the central to peripheral clocks via neural or hormonal signals (Figure 1).^4^ Most immune cells express circadian clock genes and present a wide array of genes expressed with a 24‐h rhythm, thus generating the rhythmicity of various cellular immune functions.^63^ These cell‐autonomous rhythms encompass cytokine production, trafficking, and phagocytosis.^64^, ^65^ A wide range of immune cells including macrophages, neutrophils, eosinophils, basophils, mast cells, monocytes, dendritic cells, and lymphocytes (B cells, T cells, and innate lymphoid cell [ILCs]) in diverse tissues are under the control of circadian clock (Figure 2). Circadian regulation of immune activity involves the daily fluctuation of circulating leukocyte counts, levels of secreted cytokines, tissue infiltration of immune cells, innate and adaptive immune responses, and inflammatory signaling pathways (Figure 2).

### 2.1 Macrophages

**FIGURE 3:**
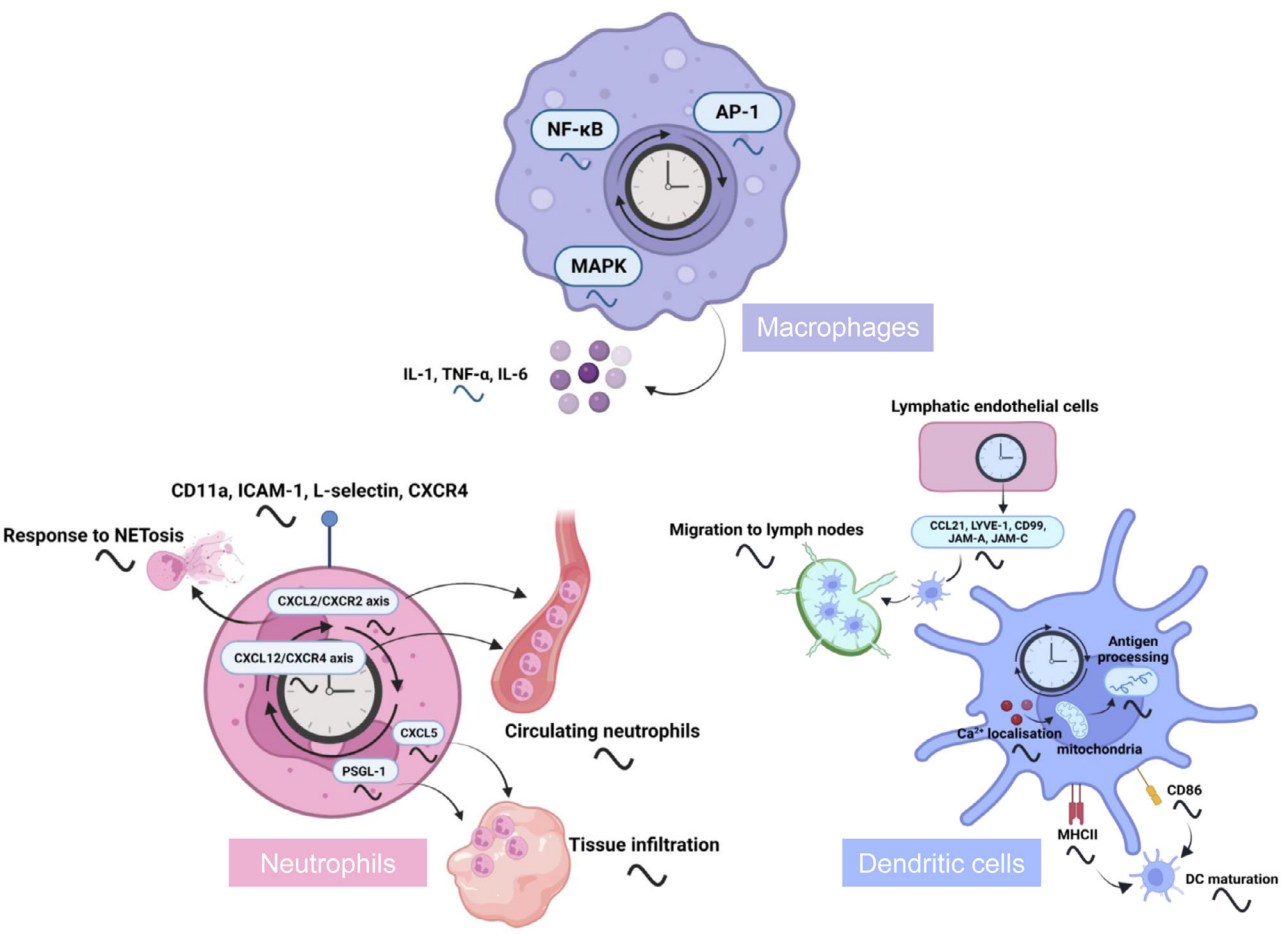
Regulation of myeloid cell behaviors and functions by the circadian clock. The circadian clock orchestrates the rhythmic patterns in various aspects of myeloid cell activities, encompassing their circulation, recruitment to lymph nodes, tissue infiltration, maturation, as well as the synthesis and release of cytokines, antigen processing, and subsequent immune responses. Circadian rhythms are orchestrated via distinct pathways, notably the CXCL2/CXCR2 and CXCL12/CXCR4 axes, and are subject to modulation by regulators such as CXCL5, PSGL‐1, CD86, and MHCII. Furthermore, a variety of regulatory elements, including NF‐κB, AP1, and MAPK, are instrumental in the precise modulation of myeloid cell behaviors and functions.Notably, DCs are categorized into two developmental lineages: a myeloid lineage, shared with phagocytes, and a lymphoid lineage, shared with T cells. AP‐1, activator protein‐1; CCL, CC chemokine ligand; DC, dendritic cell; ICAM‐1, intercellular adhesion molecule‐1; JAM, junctional adhesion molecule; LYVE‐1, lymphatic vessel endothelial hyaluronan receptor 1; MAPK, mitogen‐activated protein kinase; MHC, major histocompatibility complex; PSGL‐1, P‐selectin glycoprotein ligand‐1.

Macrophages possess a robust circadian clock that plays a crucial role in driving their inflammatory and immune functions (Figure 3).^66^ About 8% of macrophage transcriptome including clock factors, inflammatory cytokines, and regulators of inflammatory cytokines such as pathogen recognition receptors and inflammatory signaling pathways oscillates in a circadian manner.^67^ A previous study identified 1403 genes, which account for 8.1% of the total, to be rhythmically expressed in a circadian manner.^68^ Another study involving transcriptome and proteome profiling demonstrates that only 15% of circadian proteome show corresponding oscillating mRNA patterns in murine bone marrow‐derived macrophages, suggesting that post‐transcriptional regulation plays a significant role in affecting the clock regulatory output in macrophages.^69^ This observation may be attributed to the abundance of proteins involved in degradation and translation. Macrophages exhibit unique circadian post‐transcriptional regulation due to their need for prompt response to a wide range of potential immunological stimuli.^69^

Clock genes, cytokines, and chemokines exhibit robust circadian rhythms in macrophages （Figure 3）.^66^, ^70^ Clock genes such as *Rev‐erbα* and *Per2* display rhythmic expressions with high‐amplitude diurnal oscillations in peritoneal macrophages and central nervous system resident macrophages (microglia).^67^, ^71^ Tumor necrosis factor (TNF)‐α and interleukin (IL)‐6 secretion are observed in lipopolysaccharides (LPS)‐treated spleen macrophages with a peak phase around the transition time from light to dark, suggestive of a circadian clock‐regulated macrophage‐dependent cytokine secretion.^67^ Pro‐inflammatory cytokines show higher levels during the light phase in hippocampal microglia.^72^ Disruption or attenuation of core clock genes often disturbs macrophage circadian behaviors or functions. For example, circadian disruption induced by chronic jet lag modifies the rhythmic patterns of M1/M2 macrophages and cytokine levels in the spleen and tumor tissues.^73^ Deficiency of *Rev‐erbα* abolishes the circadian rhythmicity of IL‐6 in LPS‐induced macrophages and endotoxin‐treated mice.^74^ *Bmal1* deficiency decreases pro‐inflammatory cytokines (e.g., IL‐1β, TNF‐α, and IL‐6) and increases anti‐inflammatory cytokines (e.g., IL‐10) in microglial BV2 cells following LPS stimulation.^75^ In glioblastoma, deletion of *Clock* or its target gene *OLFML3* (a microglia‐attracting chemokine) decreases intratumoral microglia infiltration in the tumor microenvironment and increases overall survival.^76^ Pathogen recognition receptors, a group of innate immune receptors, serve as upstream regulators of cytokines/chemokines such as IL‐1, TNF‐α, and IL‐6 through shared signaling modules including NF‐κB, activator protein‐1 (AP‐1), and MAPK,^77^ and regulation of NF‐κB, AP‐1, and MAPK by circadian clocks may contribute to the diurnal variations of cytokines/chemokines.^78^, ^79^, ^80^

### 2.2 Neutrophils

Neutrophils are the predominant leukocyte subset in blood. The numbers, behaviors, and functions of neutrophils show apparent diurnal oscillations (Figure 3). The infiltration of neutrophils displays differential circadian rhythmicity in specific subsets and tissues.^81^ Under steady‐state conditions, neutrophil counts in the blood show circadian fluctuations.^82^, ^83^ In both humans and mice, peripheral neutrophil numbers decrease during the rest phase, whereas their numbers increase during the active phase.^84^ The levels of CD62^−^ CXC‐chemokine receptor (CXCR) 4^+^ neutrophils in circulation also follow a circadian rhythm with a nadir in the early active phase in mice. Consistently, blood neutrophil counts are lowest in the early morning in the UK Biobank participants.^83^, ^85^ This is because the release of young neutrophils and clearance of aged neutrophils from the periphery exhibit a time‐dependent manner.^83^, ^85^

Neutrophil homing to most naïve tissues such as lung and heart exhibits diurnal variations with a peak at night, whereas infiltration of neutrophils shows no circadian rhythms in the intestine, liver, and white adipose tissue.^86^ The ability of neutrophils to form neutrophil extracellular traps (NETs) also follows a circadian oscillation. In humans, the capacity of granule‐loading and NETs formation in neutrophils are higher at around 8:00 a.m. and gradually decrease later in the day.^87^ In mice, neutrophils express more enhanced levels of proteins related to granules, NETs generation, and cell migration in the nighttime.^88^ Specific deletion of *Bmal1* abolishes daily variations in granule contents and NETs formation.^89^ Some markers such as CD11a, intercellular adhesion molecule‐1, L‐selectin, and the chemokine receptor CXCR4 in neutrophils exhibit circadian oscillations in humans, while P‐selectin glycoprotein ligand‐1 (PSGL‐1), L‐selectin, CD11a, and CD29 display rhythmic expression in mice (Figure 3).^81^ Antagonistic chemokine signaling drives neutrophil release from bone marrow through two main receptors CXCR4 and CXCR2. BMAL1 coordinates diurnal changes in the transcriptional and migratory properties of circulating neutrophils by regulating chemokine CXCL2 and chemokine receptor CXCR2 (Figure 3).^90^ Genetic disruption of the circadian clock has an impact on neutrophil trafficking. CXCL12/CXCR4 axis controlled by the central clock underlies the circadian variations of neutrophil numbers in blood (Figure 3). Genetic deletion of *Cxcr4* in myeloid cells leads to a blunted oscillation of neutrophil infiltration into the blood.^91^ Downregulation of CXCL12 in the daytime stimulates the egress of neutrophils from the bone marrow into the blood.^92^ In addition, ablation of *Bmal1* in myeloid cells leads to a lack of rhythmic donor neutrophil migration into the spleen, which is attributed to a reduced PSGL‐1 (an adhesion molecule involved in immune cell trafficking) expression.^93^

### 2.3 Dendritic cells (DCs)

DCs play a pivotal role in the adaptive immune system, orchestrating antigen phagocytosis, processing, and presentation to naïve T cells, thereby initiating immune responses in pathological settings. The intricate orchestration of this process involves the expression of core clock genes (i.e., *Per1*, *Per2*, *Bmal1*, and *Clock*) and CCGs within DCs, which exhibit inherent circadian rhythmicity (Figure 3).^94^ Synchronized DCs demonstrate robust oscillations in the expressions of *Bmal1, Rev‐erbα*, and *Per2*.

The circadian rhythm regulates the differentiation, behaviors, and functions of DCs. First, loss of *Rev‐erbs* increases the expressions of surface molecules and genes (i.e., major histocompatibility complex II [MHCII] and CD86) that are important for DC maturation and immune responses. Besides, *Rev‐erb*α deficiency shows no effects on CD11b^+^CD11c^+^ DC populations, whereas *Rev‐erbβ* deficiency increases the percentage of CD11b^+^CD11c^+^ DC cells, suggesting a unique role of *Rev‐erbβ* in CD11b^+^CD11c^+^ DC development.^95^ In addition, the circadian rhythm of antigen processing in DCs is noticeably disrupted upon deletion of *Bmal1*. These findings collectively highlight the substantial contribution of molecular clock in governing the antigen‐processing capabilities of DCs.^96^ Furthermore, migration of DCs into lymph nodes also exhibits a circadian pattern, which peaks during the rest phase in mice (Figure 3).^82^ Circadian‐regulated functionality of DCs intricately shapes adaptive immune responses. For instance, intravenous administration of ovalbumin‐loaded DCs into mice in the daytime prominently augments the population of CD8+ T cells within the spleen, compared to nighttime administration.^96^

Biological clocks have emerged as crucial regulators of the trafficking DCs into lymph nodes. First, circadian clock genes are essential for maintaining the rhythmic migration of DCs into lymph nodes, involving both lymphatic endothelial cells (LECs) and DCs. Circadian expression of factors such as CC chemokine ligand 21 (CCL21), lymphatic vessel endothelial hyaluronan receptor 1, CD99, junctional adhesion molecule (JAM)‐A, and JAM‐C, which are associated with DC migration, is found to be dependent on the presence of functional BMAL1 in LECs. Notably, rhythmic expression of CCR7, a receptor for CCL21, is directly modulated by BMAL1 in DCs (Figure 3).^97^ BMAL1 also plays a significant role in DC antigen processing by influencing Ca^2+^ localization, mitochondrial dynamics, and metabolic processes, thereby impacting adaptive immune responses.^96^ Additionally, REV‐ERBα has been identified as a contributor to DC development, as it enhances the expression of MHCII and the co‐stimulatory receptor CD86, both of which serve as markers for DC maturation (Figure 3).^95^

### 2.4 T cells

**FIGURE 4:**
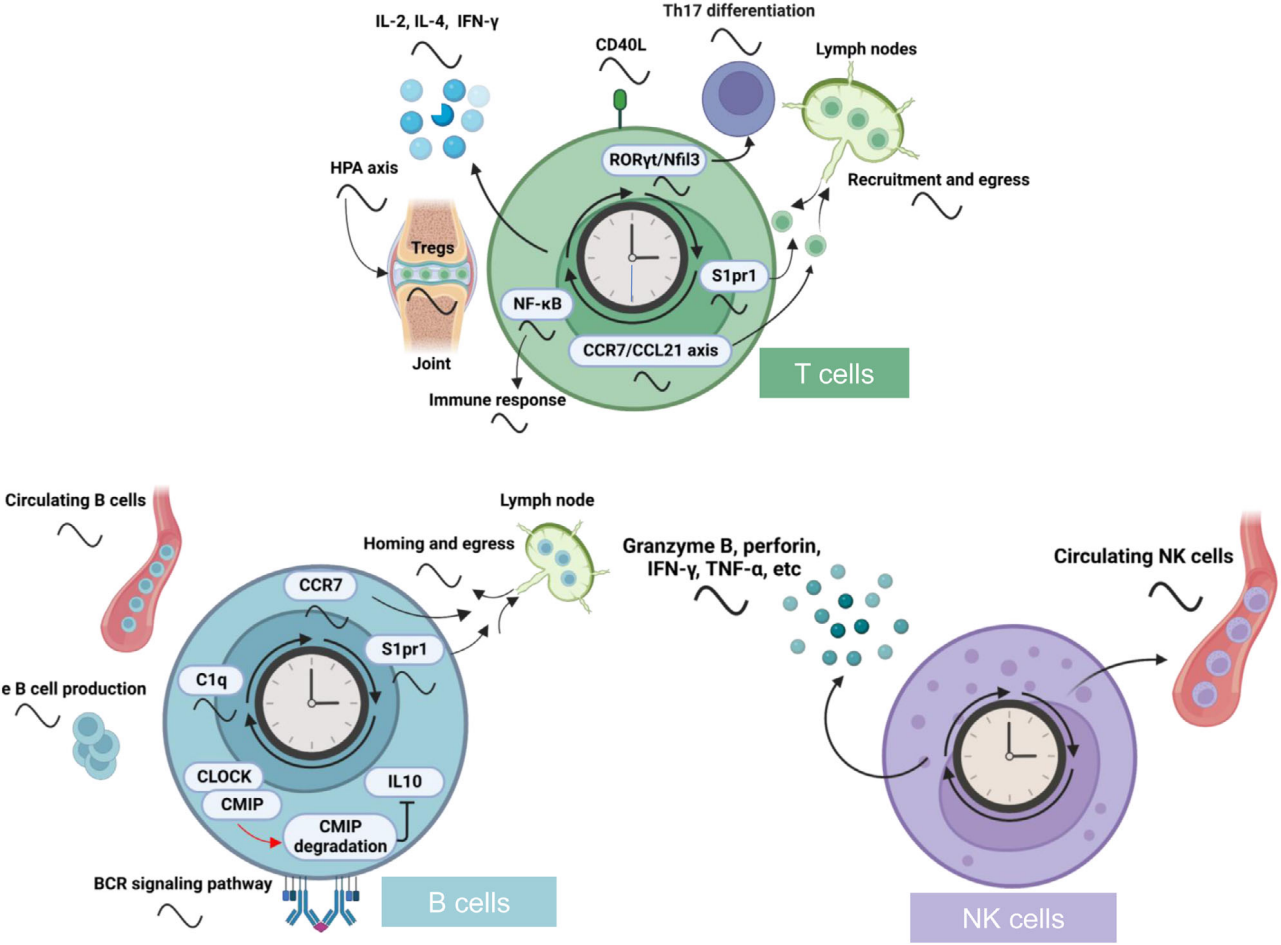
Regulation of lymphocyte behaviors and functions by the circadian clock. Circadian rhythms of lymphocyte behaviors and functions are regulated by the circadian clock at diverse aspects such as circulation of lymphocyte populations, recruitment, and egress to lymph nodes, tissue infiltration, cell differentiation, and maturation, as well as the release of cytokines, perforin, granzyme B and immune responses. The rhythmicity of these behaviors and functions are driven by clocks via different pathways such as the CCR7/CCL21 axis, CMIP, NF‐κB, S1pr1, and C1q. BCR, B‐cell receptor; CCR, CC chemokine receptor; CMIP, c‐Maf inducing protein; Nfil3, nuclear factor interleukin‐3; NK, natural killer; RORγt, retinoid‐related orphan receptor gamma t; S1pr1, sphingosine 1‐phosphate receptor 1; T_H_17, T helper 17.

Lymphocytes, as a prominent subset of immune cells responsible for immune recognition, exhibit significant circadian alterations in their numbers, including granulocytes and lymphocytes (Figure 4). T lymphocytes, a principal subset of lymphocytes, are identifiable through surface markers such as CD3, CD4, and CD8. These cells, upon activation, initiate a process of proliferation and differentiation, culminating in the secretion of cytokines, notably interferon (IFN)‐γ and IL‐2. Diverse subtypes of T cells, notably within the CD4+ and CD8+ subsets, display circadian rhythms. The number of antigen‐stimulated IFN‐γ+CD4+ T cells is higher during the nighttime, while their proportion significantly decreases during the daytime.^98^ CD4‐positive T cells exhibit pronounced circadian oscillations in various parameters, including clock gene expressions, cytokine release (IL‐2, IL‐4, IFN‐γ), and CD40L expression post stimulation (Figure 4).^98^ Natural regulatory T cells (T_reg_), crucial in shaping adaptive immune responses, exhibit a clear circadian rhythm, with their highest levels occurring at night and lowest during the day.^99^ Additionally, molecular clocks within T cells play a role in regulating their trafficking behaviors, including homing and egress within lymph nodes.^100^ Circadian clocks also participate in the regulation of T‐cell differentiation. It has been established that molecular clocks, such as BMAL1, CLOCK, and REV‐ERBα, directly govern the differentiation of T helper 17 (T_H_17) cells.^101^

The rhythmic oscillations of T‐cell numbers, reactivity, functions, and trafficking behaviors are reliant on biological clocks or circadian cues. To begin with, the circadian rhythm of T‐cell numbers is primarily affected by endogenous cortisol production, characterized by higher concentrations in the early morning and lower concentrations at midnight. This cortisol production is under the control of the circadian clock (Figure 4).^102^ Recruitment of T cells to lymph nodes is determined by the rhythmicity of T cells themselves as well as the microenvironment, which are regulated by CCR7–CCL21 axis. Additionally, oscillatory expression of sphingosine 1‐phosphate receptor 1 (S1pr1), controlled by BMAL1 and CLOCK, governs the rhythmic egress of T cells (Figure 4).^101^ Moreover, NF‐κB pathway influenced by the cell‐intrinsic clock has been identified as a potential mechanism mediating the rhythmicity of T‐cell immune responses (Figure 4).^100^ Furthermore, diurnal variations in T‐cell function are influenced by the circadian oscillation of DC function, which is regulated by the biological clock.^96^ Last, ablation of *Clock* has been found to diminish the capacity for T_H_17‐cell differentiation and reduce T_H_17‐cell frequencies in the gut through the regulation of *retinoid‐related orphan receptor gamma t* (*Rorγt*) transcription and *Nfil3* expression (Figure 4).^102^

### 2.5 B cells

B cells, as a crucial component of the adaptive immune system, display diurnal variations in their numbers. In human blood, B‐cell numbers exhibit a significant peak during nighttime, followed by decreased levels during the daytime.^103^ Additionally, B‐cell counts in lymph nodes also display rhythmicity, with the highest levels during the early dark phase, which is closely associated with circadian behaviors such as homing and egress (Figure 4).^101^ Moreover, mRNA levels of canonical clock genes, including *Bmal1*, *Clock*, and *Per2*, exhibit oscillations in splenic B cells under the daily light–dark cycle and constant conditions.^104^ B‐cell receptor (BCR) signaling pathway plays a critical role in the activation, proliferation, and differentiation of B cells. Notably, loss of CRY, a circadian protein, leads to a significant activation of BCR‐proximal signaling pathways and affects B‐cell functions.^105^ Hence, circadian clock plays a vital role in modulating B cell numbers, development, and functions.

Regulation of B‐cell numbers, development, and functions relies on both circadian clocks and external cues. First, the diurnal rhythm in circulating B‐cell numbers in humans is influenced by the rhythmic plasma cortisol levels. This diurnal oscillation of B‐cell numbers coincides inversely with plasma cortisol levels.^106^ Second, the biological clock also plays a crucial role in modulating B‐cell numbers. Genetic deletion of clock proteins has been shown to impair B‐cell development and leads to a significant reduction in B‐cell populations in peripheral blood, spleen, and bone marrow.^107^ Additionally, loss of *Bmal1* specifically in B cells disrupts the overall oscillation of B‐cell numbers in lymph nodes by regulating the diurnal expressions of key regulators such as Ccr7 and S1pr1, which are essential for lymphocyte trafficking (Figure 4).^101^ Moreover, CLOCK forms a complex with c‐Maf inducing protein (CMIP) to induce the degradation of CMIP, resulting in restricted transcription of the *Il‐10* gene in B cells. This, in turn, suppresses the immune regulatory functions of B cells (Figure 4).^28^

### 2.6 ILCs

The ILC family is stratified into two principal subtypes: cytotoxic and helper‐like ILCs. The cytotoxic subset encompasses natural killer (NK) cells, renowned for their inherent ability to target and eliminate malignant or pathogen‐infected cells. Conversely, the helper‐like ILCs are segregated into three primary groups: ILC1, ILC2, and ILC3. Each group exhibits distinct functional characteristics and plays a pivotal role in orchestrating immune responses.

NK cells, a crucial subset of lymphocytes distinguished by the absence of the CD3‐T cell receptor (TCR) complex, play a pivotal role in safeguarding the host against microbial infections and malignant transformations by means of discharging cytolytic granules or TNF‐α and triggering the Fas pathway.^108^ Recent studies have elucidated that within NK cells, the expression patterns of core circadian genes, including *Bmal1*, *Clock*, *Per1*, and *Per2*, display a distinct circadian rhythm. This rhythmic expression extends to cytolytic granules, notably granzyme B and perforin, as well as NK cell‐associated cytokines such as IFN‐γ and TNF‐α. These findings indicate a time‐dependent modulation of NK cells’ cytolytic activity, underpinned by the circadian oscillation of both gene expressions and effector functions (Figure 4).^109^ Additionally, circulating NK cell counts undergo a 24‐h oscillation in humans, displaying peak levels during the early morning and diminishing during the night (Figure 4).^110^ Nevertheless, the underlying mechanism responsible for the diurnal variations in NK cell numbers remains elusive.

Disruption of clock genes through genetic manipulation or exposure to environmental factors has provided valuable insights into the distinct roles of biological clocks in regulating the functions of NK cells. For instance, specific knockdown of *Per2* using RNA interference techniques causes a significant decrease in the levels of granzyme B and perforin within the NK cell line RNK16.^111^ Additionally, loss of *Per1* alters the rhythmic levels of perforin, granzyme B, and IFN‐γ released by splenic NK cells (Figure 4).^112^ Furthermore, chronic shift‐lag condition accelerates the aging process of NK cells, characterized by a reduced expression of Ly49 and impaired releases of granular CD107a and IFN‐γ.^113^ Repeated phase adjustments in the light–dark cycle similarly disrupt the rhythmic expressions of clock genes, cytolytic factors, and cytokines in NK cells, thereby impeding their time‐dependent cytolytic activities.^114^

In comparison to ILC1 and ILC2, ILC3 exhibits higher expressions of a range of clock genes, such as *Bmal1*, *Clock*, *Nfil3*, *Dbp*, *Pers*, *Crys*, and *Rev‐erbs*, which shows more distinct rhythmicity.^23^ The interplay between circadian clocks and ILC3 has garnered increasing attention in recent research. External cues like light exposure, dietary patterns, and microbiota composition, along with intrinsic circadian mechanisms, have been identified as modulators of the rhythmic expressions of clock genes (i.e., *Clock* and *Rev‐erbα*) in ILC3.^23^ Furthermore, circadian clock exerts a regulatory influence over the quantity, behavior, and functional attributes of ILC3. Notably, ablation of *Bmal1* leads to a marked decrease in the population of ILC3 within the small intestine of mice.^115^ REV‐ERBα, a pivotal transcription factor for ILC3, significantly influences various ILC3 subsets.^116^ Perturbations in circadian rhythm have been observed to impact cytokine secretion in ILC3. For instance, Rev‐erbα deficiency substantially diminishes the numbers of NKp46+ ILC3 cells, reduces RORγt expression, and lowers IL‐22 production, while paradoxically elevating IL‐17 secretion.^116^ In ILC3, BMAL1 deficiency shows enhanced expressions of RORγt‐dependent target genes, including *IL17a*, *IL17f*, and *IL22*.^115^ Investigations into various types of ILCs, beyond ILC3, remain relatively sparse. However, notable advancements have been made in understanding the role of RORα in the differentiation of ILC2. These cells are integral to type II inflammation, allergic responses, and defense mechanisms against parasitic infections. The significance of RORα in the differentiation process of ILC2 underscores the critical functions of these cells in the immune response.^61^ Additionally, ILC2, which is under circadian regulation, play a vital role in the maintenance of eosinophil homeostasis.^117^

The circadian regulation of helper‐like ILCs is governed by a multitude of mechanisms. BMAL1 deficiency leads to a decrease in ILC3 counts in the small intestine, and an increased presence of ILC3s in the mesenteric lymph nodes attributed to loss of gut homing signals such as CCR9, α4β7 integrin, and CXCR4.^23^ Furthermore, gut microbiota and pro‐apoptotic proteins, such as Bim and Bax, are identified as key factors in controlling the hyper‐activation and loss of intestinal ILC3 in BMAL1‐deficient mice. The elimination of microbiota through antibiotic treatment partially mitigates the hyper‐activation of *Bmal1*‐deficient ILC3 and restores cellular homeostasis in the gut.^115^ However, regulatory mechanisms of helper‐like ILCs by circadian clocks have remained largely unexplored.

## 3 CIRCADIAN REGULATION OF NON‐IMMUNE CELL FUNCTION

Circadian oscillations manifest within a diverse array of non‐immune cellular populations. Our investigative endeavors have been profoundly directed toward three distinct categories of rhythmically orchestrated cell types, namely, hepatocytes, adipocytes, and intestinal epithelial cells, all of which assume pivotal roles in physiological processes. Hepatocytes, which constitute the majority of liver mass, are central to an array of biochemical and metabolic functions.^118^ Adipocytes, along with their secretory products, are involved in numerous physiological processes and significantly influence metabolic health.^119^, ^120^ Intestinal epithelial cells, essential for food digestion, nutrient absorption, and maintaining intestinal epithelial homeostasis, play a vital role in responding to diseases.^121^ A substantial body of evidence demonstrates that circadian clock genes are intricately involved in the physiological functions of these cells.

### 3.1 Hepatocytes

Hepatocytes, as the principal parenchymal cells in the liver, orchestrate a myriad of cellular processes including drug metabolism, drug detoxification, and immune cell activation, all critical for preserving liver homeostasis.^118^ The circadian clock mechanism exerts regulatory control over the diurnal rhythmicity of gene expressions and functional processes within hepatocytes. Perturbation of REV‐ERBs disrupts the natural diurnal rhythm and induces alterations in the metabolomic profiles observed across diverse liver cell categories.^122^ Furthermore, disruption of CLOCK function results in modified diurnal patterns of cytochrome P450 enzymes within hepatocytes, including but not limited to CYP2A4/5 and CYP2B10.^123^

The hepatocyte clock plays a vital role in sustaining rhythmic gene expression patterns in immune cells. Specifically, hepatocyte‐specific knockout of *Rev‐erbs* leads to marked changes in the rhythmic gene expression profiles of nonparenchymal liver cells, with macrophages being notably affected.^124^ Hepatocyte‐targeted *Rev‐erbs* knockdown disrupts the rhythmicity of genes linked to the vitamin D receptor signaling pathway, a pathway known to mitigate liver inflammation and steatosis. It also enhances the circadian rhythmicity of genes associated with inflammatory responses in macrophages.^124^, ^125^, ^126^ The interplay between hepatocytes and macrophages, facilitated by mediators including hepatocyte‐derived sterol regulatory‐element binding protein (SREBP) cleavage‐activating protein and a range of polypeptides, plays a critical role in the transmission of circadian rhythmic signals.^124^ In conclusion, circadian clocks are integral to the regulation of gene expressions and cellular functions in hepatocytes, underscoring their role in maintaining hepatic and systemic physiological balance.

### 3.2 Adipocytes

Adipose tissue dynamically responds to the daily fluctuations in energy availability and demand, a crucial aspect of maintaining energy homeostasis. This adaptability is facilitated by a circadian modulation of signals, where adipose physiology is governed in a time‐of‐day dependent manner. This rhythmic regulation is primarily achieved through the transcriptional control of CCGs, which are regulated by adipocyte clocks.^127^ Adipocyte clocks orchestrate the timing of the local transcriptome, which includes critical genes involved in lipogenesis and lipolysis, such as *Atgl*, *Hsl*, *caveolin 2*, *acyl‐CoA synthetase*, *phosphatidate phosphatase*, *Lpl*, *peroxisome proliferator‐activated receptor α/γ*, *coactivator 1β*, and *Srepb1α*.^127^

The BMAL1/CLOCK heterodimer in adipocyte assumes a central role in the regulation of gene expressions, specifically activating the transcription of *Atgl* and *Hsl* genes by binding to E‐box promoter elements.^128^ Notably, adipocyte‐selective deletion of Rev‐erbα, under normal conditions, exhibits a relatively modest impact on the expressions of lipogenic genes. However, in the context of diet‐induced obesity, this deletion exerts a profound influence on metabolic health. It leads to the development of a metabolically advantageous phenotype characterized by diminished adipose tissue inflammation and distinct alterations in the dynamics of collagen fibrosis. These effects are attributed to the regulatory control of collagen genes.^129^ Furthermore, circadian clock exerts temporal control over adipogenic differentiation. BMAL1 transcriptionally regulates genes associated with the actin dynamic‐MRTF/SRF cascade, thereby influencing actin cytoskeleton organization and SRF activity. Additionally, circadian clock exerts precise temporal control over adipogenic differentiation. BMAL1 orchestrates the transcriptional regulation of genes associated with the actin dynamic‐myocardin‐related transcription factor/serum response factor (MRTF/SRF) cascade, thereby influencing the organization of the actin cytoskeleton and modulating SRF activity. This regulatory mechanism holds pivotal importance in the transformation of adipogenic progenitor cells into mature adipocytes, the process of beige adipogenesis, and the enhancement of thermogenic capacity.^130^ In summary, circadian clocks play a vital and multifaceted role in both the functions and differentiation of adipocytes, underscoring their significance in the realm of adipose tissue physiology and the broader context of systemic energy homeostasis.

### 3.3 Intestinal epithelial cells

Intestinal epithelial cells, a single layer of tightly interconnected columnar cells, form the primary defensive barrier of the intestinal mucosa.^131^ Circadian clock regulates cellular processes in intestinal epithelial cells including growth, proliferation, differentiation, and cell signaling.^132^ Of particular note, BMAL1 plays a central role in the regulation of intestinal epithelial cell regeneration by modulating cytokine activity, cell cycle progression, and cell proliferation dynamics. It is worth highlighting that knockdown of *Bmal1* results in the disruption of the rhythmic pattern of cellular proliferation.^133^, ^134^ Furthermore, TNF cytokine signaling and P21 emerge as key contributors to BMAL1‐mediated cell proliferation control. The former fosters proliferation among epithelial precursors, thereby facilitating the replacement of damaged cells, while the latter assumes a pivotal role in regulating diurnal rhythms in cell production.^134^ The epithelial clock also exerts regulatory control over essential molecules such as Wee1, cyclins D1, and E, all of which are indispensable for the generation and maintenance of circadian rhythms in the cell cycle progression of intestinal epithelial cells.^135^ Additionally, intestinal stem cells, vital for the continuous replenishment of differentiated epithelial cells, are influenced by clock components including BMAL1 or CRY. These components modulate transcripts linked to regeneration and stem cell signaling, encompassing pathways like Wnt and Hippo signaling within the intestinal epithelium.^136^

The circadian clock serves as a key regulator in managing both endogenous substances and xenobiotics within intestinal epithelial cells, highlighting its essential role in cellular processing and homeostasis. Intestinal BMAL1 transcriptionally enhances DGAT2 expression, an enzyme crucial for triacylglycerol synthesis, by binding to an E‐box in its promoter, thereby facilitating dietary fat absorption in the gut.^137^ Intestinal exporter MRP2, which plays a key role in the disposition and elimination of various drugs, is also impacted by the intestinal circadian clock. Loss of *Bmal1* in intestinal epithelial cells diminishes the expression and transport activity of MRP2, as well as their diurnal rhythms, affecting the pharmacokinetics and toxicity of medications like methotrexate.^138^

## 4 CIRCADIAN REGULATION OF CELLULAR SIGNALING PATHWAYS

The circadian clock is crucial in regulating signaling pathways via transcriptional and translational modulation.^13^NF‐κB, JAKs/STATs, and MAPKs signaling pathways, central to inflammatory responses and diverse diseases, are significantly influenced by circadian rhythms.^42^ The following sections critically examine the mechanistic connections between the circadian molecular clock and these signaling pathways: NF‐κB, JAKs/STATs, and MAPKs.

### 4.1 NF‐κB signaling pathway

**FIGURE 5:**
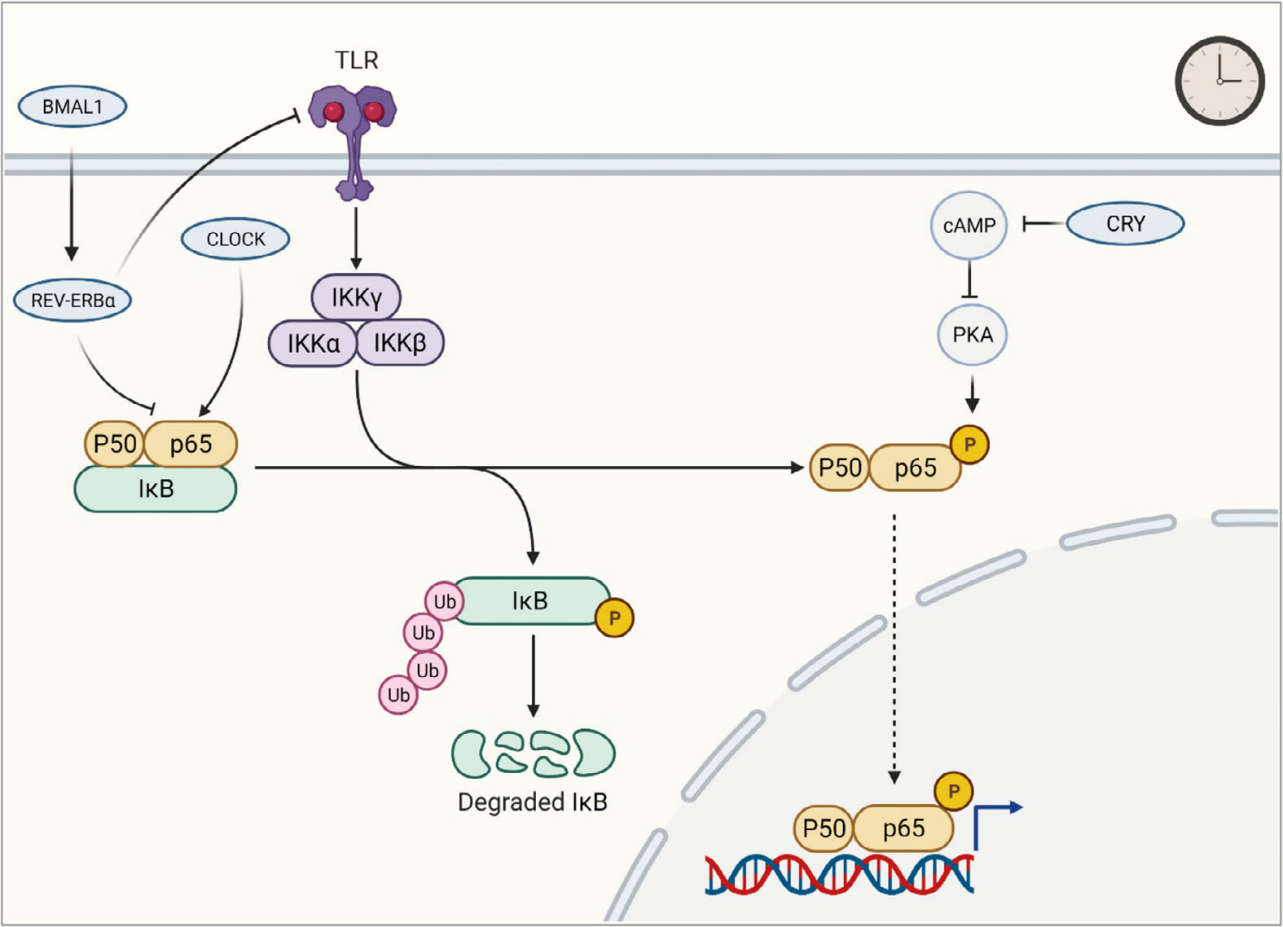
Regulation of NF‐κB signaling pathway by the circadian clock. Circadian clocks regulate NF‐κB signaling pathway through diverse aspects including transcription, post‐translational modification, and protein–protein interactions. REV‐ERBα regulates NF‐κB via modulation of p65 subunit and TLR4. BMAL1 acts as an NF‐κB/NLRP3 repressor through REV‐ERBα. CLOCK interacts with the p65 subunit. CRY1 binds to adenylyl cyclase to limit cAMP production and inactivates PKA, subsequently resulting in NF‐κB activation through phosphorylation of p65. cAMP, cyclic adenosine monophosphate; CLOCK, circadian locomotor output cycles kaput; CRY, cryptochrome; IKK, IκB kinase; NF‐κB, nuclear factor‐κB; PKA, protein kinase A; cAMP, cyclic adenosine monophosphate; REV‐ERBα, nuclear reverse erythroblastosis virus heme receptor α; TLR, Toll‐like receptors.

NF‐κB signaling pathway, comprising a family of inducible transcription factors, is a pivotal regulator of inflammatory responses.^140^ Circadian clock modulates various aspects of the NF‐κB signaling pathway targets (Figure 5).^141^ Clock factors form an intricate network with NF‐κB proteins, influencing their function and interaction. REV‐ERBα, a key clock component, regulates the levels of p65, a subunit of NF‐κB, orchestrating the expressions of inflammatory genes and the release of cytokines and chemokines through three distinct mechanisms. First, Rev‐erbα suppresses the Toll‐Like Receptor 4, thereby blocking upstream pro‐inflammatory signals in the NF‐κB pathway.^142^ Second, REV‐ERBα disminishes the nuclear level of p65 protein, inhibiting its transcriptional activation of target genes. Third, REV‐ERBα directly binds to the promoters of inflammatory genes (e.g., [NOD‐, LRR‐ and pyrin domain‐containing protein 3]*Nlrp3*, *Il‐1β*, and *Il‐6)*, suppressing their transcription and expressions. Multiple mechanisms are proposed for the regulation of NF‐κB signaling by the clock protein BMAL1. BMAL1 regulates NF‐κB/NLRP3 axis via REV‐ERBα, which acts as a repressor of NF‐κB/NLRP3. Supporting this, BMAL1 influences the timing of gene expression in response to inflammatory activation by modulating the epigenetic status of enhancers, partly through the regulation of REV‐ERBs and eRNA transcription in macrophages.^143^ Biochemical and biophysical analyses have shown that p65 subunit of NF‐κB binds to the transactivation domain of BMAL1 through protein–protein interaction,^144^ with additional mechanisms including an increase in the phosphorylation of IκB.^144^ CLOCK directly interacts with p65 protein, and CLOCK overexpression leads to an increase in specific phosphorylated and acetylated transcriptionally active forms of p65.^145^ CRY1, another clock component, binds to adenylyl cyclase, limiting cAMP production and inactivating protein kinase A, which results in NF‐κB activation through phosphorylation of p65 at S276.^146^ These studies provide a comprehensive mechanistic framework for understanding the intricate relationship between the circadian clock and NF‐κB signaling.

### 4.2 MAPK signaling pathway

MAPK signaling pathways are composed of three families including extracellular‐signal‐regulated kinase (ERK), c‐Jun NH2‐terminal kinase (JNK), and p38. Clock factors physically and/or genetically interact with MAPKs to regulate inflammatory responses. BMAL1 decreases ERK phosphorylation in chondrocytes^147^ and inactivates p38 phosphorylation after traumatic brain injury.^78^ REV‐ERBα suppresses osteoblast differentiation by inhibiting the p38 MAPK pathway,^148^ while ROR‐γ‐knockdown downregulates p38 MAPK expression in hepatocytes.^149^ These regulatory mechanisms are attributed to circadian rhythms in the expression of MAPK genes and/or proteins such as ERK and MAPKs (upstream of p38 and JNK kinases).^150^

### 4.3 JAK‐STAT signaling pathway

JAK‐STAT signaling pathway is critical for orchestrating the immune system. The circadian clock modulates JAK‐STAT signaling pathway via two separable mechanisms. First, REV‐ERBα abrogates JAK/STAT3 signaling pathway as evidenced by reduced phosphorylated JAK1, JAK2, and STAT3. This effect was attained by up‐regulating the suppressor of cytokine signaling 3, an inhibitor of the JAK/STAT3.^151^ In addition to the core clock genes, miR‐279 acting as an effector of clock‐controlled behavioral output, targets *Upd* (the ortholog of the JAK/STAT ligand) to circadian behavioral output.^152^ Consequently, STAT92E and phosphorylated HOP (JAK/STAT components) are expressed in a circadian manner.^153^ The regulation of JAK/STAT3 by circadian clock bears significance for the etiology of inflammatory and autoimmune disorders.

## 5 CIRCADIAN REGULATION OF DISEASES

**FIGURE 6:**
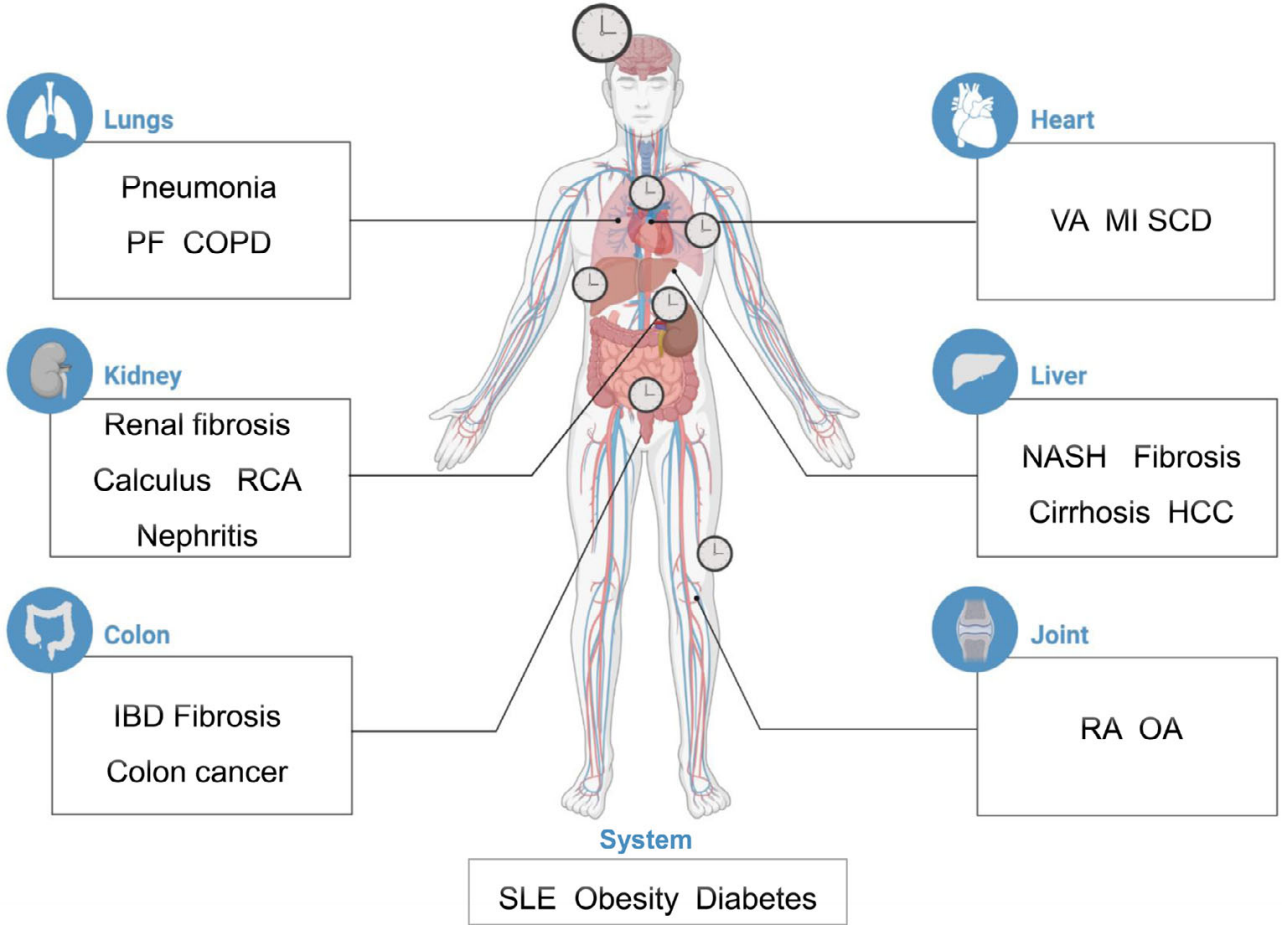
The role of the circadian clock in diseases. Circadian clocks situated in the lung, kidney, colon, heart, liver, and joint tissues exert regulatory effects over diseases. COPD, chronic obstructive pulmonary disease; HCC, hepatocellular carcinoma; IBD, inflammatory bowel disease; MI, myocardial infarction; NASH, nonalcoholic steatohepatitis; OA, osteoarthritis; PF, pulmonary fibrosis; RA, rheumatoid arthritis; RCA, right coronary artery; SCD, sudden cardiac death; SLE, systemic lupus erythematosus; VA, ventricular arrhythmias.

Dysregulation of circadian rhythms within distinct cellular populations stands out as a pivotal mechanistic factor contributing to a wide spectrum of pathological conditions spanning multiple tissue types. These afflictions encompass a range of conditions intimately linked to typical circadian patterns, including inflammatory diseases, cancers, and systemic diseases (Figure 6 and Table 1).^154^

**TABLE 1:** The effects of circadian disruption on cell functions in diseases.

Cell types	Models	Diseases	Findings	Refs
Microglia	*Bmal1* deficiency	Asthma	Decrease pro‐inflammatory cytokines and increase anti‐inflammatory cytokines in microglial BV2 cells following LPS stimulation	^75^
Macrophages	*Rev‐erbα* deficiency	Rheumatoid arthritis	Abolish the circadian rhythmicity of IL‐6 in LPS‐induced macrophages and endotoxin‐treated mice	^74^
Macrophages	Jet lag	Melanoma	Induce the loss or inversion of daily patterns of M1 and M2 macrophages and cytokine levels	^73^
Microglia	*Clock* deficiency	Glioblastoma	Decrease intratumoral microglia infiltration in the tumor microenvironment and increase overall survival	^76^
Neutrophils	*Rev‐erbα* deficiency	Hepatic I/R	Aggravate hepatic I/R injury and significantly increases the number of neutrophils infiltrated to I/R liver	^155^
Neutrophils	*Bmal1* deficiency	IBD	Abolish daily variations in granule contents and NETs formation	^89^
NK cells	*Per1* deficiency	Lung cancer	Disrupt and alter the rhythmic levels of perforin, granzyme B, and IFN‐γ released by splenic NK cells	^112^
NK cells	Chronic shift‐lag	Autoimmune disease	Accelerate the aging process of NK cells, characterized by reduced expression of Ly49 and impaired release of granular CD107a and IFN‐γ	^113^
NK cells	*Per2* deficiency	Non‐small cell lung cancer	Confer greater resistance to LPS‐induced endotoxic shock, which could be attributed to decreased production of IFN‐γ/IL‐1β and suppression of NK cell function	^156^
NK cells	*Bmal1* deficiency	IBD	Reduce ILC3s counts in the small intestine and increase the frequency of ILC3s in the mesenteric lymph nodes	^23^
ILCs	*Rev‐erbα* deficiency	IBD	Reduce the cell numbers of NKp46+ ILC3 subset, RORγt expression, and IL‐22 production, whereas increase IL‐17 secretion paradoxically	^116^
ILCs	*Bmal1* deficiency	IBD	Decrease in the population of ILC3 within the small intestine of mice	^115^
DCs	*Clock* deficiency	Autoimmune inflammation	Diminish the capacity for T_H_17‐cell differentiation and reduce T_H_17‐cell frequencies in the gut	^102^
T cells	*Clock* deficiency	B‐cell lymphoma	Impair B‐cell development and lead to a significant reduction in B‐cell populations in peripheral blood, spleen, and bone marrow	^107^
B cells	*Bmal1* deficiency	Lymphomas	Disrupt the oscillation of B‐cell numbers in lymph nodes	^101^

Abbreviations: Bmal1, Brain and Muscle ARNT‐Like 1; Clock, circadian locomotor output cycles kaput; DCs, dendritic cells; I/R, ischemia/reperfusion; IBD, inflammatory bowel disease; IFN‐γ, interferon‐γ; IL, interleukin; ILC3s, Group 3 innate lymphoid cells; LPS, lipopolysaccharides; NETs, neutrophil extracellular traps; NK, natural killer; Per1, period 1; Per2, period 2; Rev‐erbα, nuclear reverse erythroblastosis virus heme receptor α; RORγt, retinoic acid receptor‐related orphan receptor γt; T_H_17, T helper 17.

### 5.1 Inflammatory and immune diseases

#### 5.1.1 Inflammatory bowel disease (IBD)

IBD, a chronic affliction of the colon that significantly impacts individuals' lives, primarily comprises two types: ulcerative colitis and Crohn's disease.^157^ IBD is characterized as a chronic and recurrent inflammatory disorder of the intestinal tract. Research involving chronic colitis in mice has demonstrated a diurnal pattern in disease severity, marked by elevated levels of inflammatory factors (IL‐1β and IL‐6) during the daytime and reduced levels at night.^158^

In contrast, disruptions in circadian rhythm have been observed to intensify a pro‐inflammatory state and foster the development of IBD. Shift workers, who often endure non‐traditional work schedules leading to abnormal sleep–wake cycles, are at an increased risk for developing IBD.^159^ In mammals, deficiencies in clock genes, including *Bmal1*, *Per1/2*, and *Rev‐erbα*, are associated with heightened susceptibility to dextran sulfate sodium/trinitrobenzene sulfonicacid (DSS/TNBS)‐induced IBD. Conversely, overexpression of these genes can mitigate the severity of colon inflammation.^25^

The progression of IBD is intricately regulated by the circadian clock through various molecular mechanisms. These encompass key factors such as NLRP3 inflammasome, NF‐κB signaling pathway, and immune cell populations, notably PDL1+ B cells and CD4+ T cells.^102^, ^160^ In addition to inflammation‐related factors, non‐inflammatory factors, including genes associated with the intestinal barrier such as tight junction protein 1 and mucin 2, along with intestinal epithelial cells, play significant roles in the circadian clock‐mediated regulation of IBD. Disruption of circadian clock specifically within epithelial cells elicits a profound exacerbation in colitis severity and mortality. This adverse outcome can be attributed to an upsurge in the production of inflammatory mediators, including signal transducer and activator of transcription, as well as gut microbiota‐derived short‐chain fatty acids.^161^ This evidence underscores a significant role of circadian rhythms in the pathophysiology of IBD, highlighting the need for further research to fully understand these complex interactions.

#### 5.1.2 Rheumatoid arthritis (RA)

RA, an autoimmune disorder, is marked by chronic inflammation in the articular cartilage, joints, and surrounding tissues. The symptoms of RA exhibit significant circadian oscillations, with increased severity in joint swelling, stiffness, and pain observed during the early morning.^162^, ^163^, ^164^ This escalation correlates with a higher nocturnal release of proinflammatory cytokines such as TNF‐α and IL‐6 in the synovium and pannus.^165^ Additionally, anti‐inflammatory hormone cortisol varies in a time‐dependent manner, typically showing insufficiency in the early morning, which contributes to the circadian variation in RA symptoms.^166^

Disruptions in circadian clocks have been observed to exert a deleterious influence on the pathogenesis of RA. Extensive investigations have demonstrated that perturbations of the molecular clock, whether induced in vivo through light misalignment and genetic interventions or in vitro through pharmacological manipulation, culminate in the alteration of clock gene expression and exacerbation of joint inflammation..^139^ For instance, deficiency of *Cry1* and *Cry2*, which are pivotal core clock genes, results in a heightened inflammatory state in collagen‐induced RA murine models, thereby underscoring the significance of clock gene integrity in the regulation of RA‐associated inflammation.^167^ Conversely,RA itself can engender disturbances in sleep patterns, characterized by difficulties in falling asleep, non‐restorative sleep, and discernible alterations in the expression profiles of clock genes such as *BMAL1*, *CLOCK*, *CRY*, and PER.^168^ These bidirectional interactions between circadian rhythms and RA pathophysiology underscore the intricate relationship between the circadian system and immune regulation in the context of this autoimmune disorder.

The development of RA is regulated by circadian clocks through various mediators. These include immune cell infiltration, pro‐inflammatory cytokines (IL‐1β, IL‐6, and TNF‐α), neuroendocrine hormones (cortisol in humans and corticosterone in rodents, and melatonin), mediators of oxidative stress (e.g., p53 and Nrf2), and neurotransmitters.^169^ For instance, increased suppression of inflammation is noted during nighttime, attributed to the elevated presence of T_reg_ cells within the joints.^57^ This complex interplay highlights a significant effect of circadian rhythms on the progression and symptomatology of RA.

### 5.2 Cancers

Cancer remains a leading cause of mortality and a significant barrier to extending life expectancy. Emerging research increasingly implicates disruptions in circadian rhythms as significant factors in the onset and progression of various cancers. For instance, shift workers exhibit an approximately 10% heightened risk of developing breast cancer.^170^ Furthermore, mice with chronic circadian disruption are more likely to develop HCC (hepatocellular carcinoma), likely due to metabolic and immunologic changes.^171^ Additionally, chronic jet lag exacerbates tumor‐induced inflammation in the mouse liver and hypothalamus, which may contribute to cancer‐related cognitive impairments and an unfavorable prognosis.^172^ In a contrasting perspective, the levels of specific clock genes, namely, *PER1*, *PER2*, *PER3*, and *CRY2*, are notably diminished in cancerous tissues, compared to their noncancerous counterparts. This reduction is primarily ascribed to promoter methylation and overexpression of *EZH2*, a gene integral to epigenetic regulation.^173^

Alterations in the components of the circadian clock, including BMAL1, PER1/2, and CRY1/2, have been correlated with an increased risk of cancer development. Specifically, BMAL1 is critical in hematologic malignancies, where its inactivation has been linked to the advancement of cancers.^174^  *Bmal1* knockout mice show a heightened susceptibility to colitis‐associated colorectal cancer.^160^ Mice with hepatocyte *Bmal1* deficiency have a protective effect in high‐fat diet‐ and diethylnitrosamine‐induced hepatocellular carcinoma in mice.^175^ The repressive arms of the core circadian clock, including PER1 and PER2, are also implicated in cancer. Reduced expression of these genes has been associated with shorter survival in patients with glioma and gastric cancer.^176^ These findings highlight the intricate connection between circadian rhythms and cancer, emphasizing the need for further research to understand how circadian disruptions contribute to cancer development and progression.

Inflammation plays a pivotal role in the regulatory effects of the circadian clock on cancer pathogenesis.^177^, ^178^, ^179^ The activation, trafficking, and infiltration of immune cells into tumors are largely contingent on the release and presentation of cancer cell antigens, with inflammation being a central mediator in this mechanism. For instance, rhythmic expression of *Bmal1* regulates the numbers of antigen‐specific DCs and the volume of tumors following tumor engraftment at different time points (ZT9 or ZT21).^180^ In a melanoma mouse model, chronic jet lag leads to significant immunosuppression within the tumor microenvironment, accelerating tumor growth. This effect is attributed to the elimination of daily variations in macrophage counts and a reduced ratio of M1 to M2 macrophages in the tumor.^73^ Additionally, *Bmal1* deficiency in mice results in a decrease in Breg+ PDL1+ cell numbers among intestinal intraepithelial lymphocytes and induces the death of activated CD4+ T cells, thereby promoting the progression of colitis‐associated colorectal cancer.^160^ Chronic shift‐lag conditions disrupt both the circadian expressions of clock genes and the cytolytic activities of NK cells, potentially fostering tumor growth in the lung.^156^ Furthermore, various regulators, such as cyclin‐dependent kinases, suppressor of mother against decapentaplegic (SMAD) family member 4, chemokines, vascular endothelial growth factor A, and telomerase reverse transcriptase, have been implicated in the influence of clock genes on cancer development.^176^ Given these insights, it is crucial to explore novel therapeutic approaches targeting cancer cells, leveraging the circadian rhythmic regulation of cancer biology. Such strategies could offer more effective and tailored treatments, potentially improving patient outcomes by aligning therapy with the body's natural circadian rhythms.

### 5.3 Systemic diseases

#### 5.3.1 Systemic lupus erythematosus (SLE)

The circadian clock is fundamentally implicated in the progression of SLE, characterized by a bidirectional relationship between circadian rhythms and the disease. This association is underscored by a large‐scale population‐based cohort study involving 144,396 patients, which identifies that sleep deprivation markedly increase the susceptibility to SLE.^181^ Complementing this finding, additional research suggests that relatives of SLE patients who consistently sleep less than 7 h per night face an elevated risk of developing the disease.^182^ Moreover, genetic factors contribute to this correlation as evidenced by the association of single nucleotide polymorphisms in the *PER2* gene with both the susceptibility to and clinical manifestations of SLE.^183^ Conversely, patients with SLE, or those at risk of developing it, often experience sleep disturbances, which significantly impair their quality of life.^184^

Studies have highlighted the role of circadian clock proteins in the pathogenesis of SLE. These studies point to various factors, including immune cells, melatonin, Nrf2, anti‐nuclear antibodies, and glomerular complement precipitation, as contributors to the disease process.^105^, ^185^, ^186^ In experimental models, conditional knockout of *E4bp4* in T cells has been shown to enhance the differentiation of T follicular helper cells, thus exacerbating pristane‐induced lupus‐like diseases in mice.^187^ Additionally, loss of *Cry* results in increased mature B‐cell production, overactivation of the BCR‐signaling pathway, and downregulation of C1q expression, thereby accelerating the pathogenesis of SLE.^106^ This growing body of evidence underscores a complex interplay between circadian rhythms and immune function in the context of SLE, highlighting the need for further research to fully understand these relationships and their implications for SLE management.

#### 5.3.2 Obesity and diabetes

Immune cells, particularly macrophages, are central to the mechanisms linking circadian rhythms with inflammation‐driven metabolic disorders including obesity and diabetes. Myeloid‐specific *Bmal1* knockout increases Ly6Chi macrophage content in adipose tissue and levels of monocyte‐attracting chemokines, as well as inflammatory responses in mice subjected to a high‐fat diet.^188^ This causes increased total body adiposity and tissue weight, highlighting the role of BMAL1 in regulating obesity‐related inflammation.^188^ Similarly, bone marrow‐derived macrophages from *Per1/2* knockout mice show a higher proportion of pro‐inflammatory M1 macrophages, exacerbating systemic insulin resistance and accelerating the progression of diabetes.^74^

In addition to the role of immune cells in diabetes, intestinal epithelial cell‐specific *Bmal1* knockout can remodel diurnal hepatic metabolism by shifting to gluconeogenesis from lipogenesis during the dark phase, and elevate the production of glucose and insulin insensitivity, leading to an enhanced susceptibility of diabetes in mice.^10^ Adipocyte‐specific deletion of *Bmal1* results in obesity accompanied with reduced energy expenditure, attenuated food intake rhythm, and increased food intake via decreasing circulating polyunsaturated fatty acid concentration and affecting the levels of neurotransmitters responsible for appetite regulation in hypothalamic feeding centers.^189^ Recent advancements in chronobiology have shed light on a significant role of the hepatocyte clock in the regulation of metabolic disorders associated with inflammation. A key finding in this domain is the discovery of *Platr4*, an anti‐inflammatory long non‐coding RNA, which operates under the control of REV‐ERBα. The primary function of *Platr4* is the inhibition of the NLRP3 inflammasome, an essential factor in the body's inflammatory response. This inhibition by *Platr4* is particularly noteworthy in the context of nonalcoholic steatohepatitis, where it helps in reducing the disease's severity.^190^

Inflammation‐associated metabolic diseases disrupt circadian clocks. For example, in obese patients, activation of NF‐κB hinders the binding of BMAL1 protein to *PER2* promoter, reducing PER2 expression and thus impairing circadian clock function.^191^ By understanding and manipulating the complex interplay between the circadian clock and inflammation, novel strategies could be developed to treat obesity and diabetes.

## 6 CONCLUSION

This review provides insights into the regulatory influence of circadian clocks on cellular functions, signaling pathways, and disease processes. The modulation of cell functions and signaling pathways by the circadian clock emerges as a pivotal determinant of the rhythmic intensity observed in various diseases. A deep comprehension of the intricate interplay between cell‐intrinsic clocks and cell functions is of paramount significance for advancing our mechanistic understanding of diseases. Furthermore, this knowledge is essential for the development of innovative chronotherapeutics and targeted drugs. These interventions aim to harness the circadian regulation of cellular functions to enhance disease management strategies.

In normal life, sleep deprivation is closely associated with circadian disruption, resulting in dysregulated immune responses and increased susceptibility to diseases.^192^, ^193^, ^194^ Epidemiological studies have robustly established a significant correlation between sleep deprivation and various cancers, including breast, colorectal, and prostate cancers.^195^, ^196^, ^197^, ^198^ Sleep‐deprived models exhibit a reduction in immune cell numbers (e.g., NK cells, T cells, DCs), creating an immunosuppressive environment conducive to accelerated cancer growth.^199^ Sleep deprivation disrupts the circadian rhythm to influence inflammatory pathologies. Sleep deprivation, by disrupting circadian rhythms, also exerts an influence on inflammatory pathologies. Interventions targeting the circadian clock can modulate inflammation induced by sleep deprivation through signaling pathways.^200^ Consequently, an exploration of novel ligands targeting circadian clocks holds substantial promise for the treatment of inflammation‐related diseases triggered by sleep deprivation.

Our review sheds light on the bidirectional relationship between circadian clocks and inflammation. Disruptions in circadian rhythms amplify inflammatory responses to pathogens and exacerbate disease progression. Conversely, inflammation can influence the expression and functional dynamics of circadian clocks. A key player in this interplay is NF‐κB, which acts as a modulator of circadian rhythm through its interaction with clock genes. NF‐κB modifies circadian rhythms by binding to the regulatory sequences of clock genes, recruiting histone deacetylases for chromatin modification, and interacting with the BMAL1/CLOCK complex at E‐box sites, thereby influencing circadian transcription.^201^ The precise mechanism by which disturbances in circadian rhythms initiate inflammation (as an “egg”) and the extent to which existing inflammation (as a “chicken”) impacts these rhythms remain unclear. However, our review acknowledges the intricate interconnection between circadian rhythms and inflammation‐associated diseases. This understanding paves the way for developing targeted therapeutic strategies to effectively manage these complex interactions.

In summary, this review underscores a pivotal role of circadian rhythm disruptions in diverse diseases. This influence is primarily mediated through cell functions and signaling pathways. There exists an urgent necessity for conducting extensive research to discover innovative approaches for intervening in circadian clock‐controlled diseases. This encompasses endeavors to address disturbed circadian rhythms and optimize disease management, thereby potentially enhancing patient outcomes and quality of life.

## AUTHOR CONTRIBUTIONS

Y.L., L.H., Y.C., X.W., S.W., and F.L. conceived, edited, and wrote this manuscript. Y.L., L.H., S.W., and F.L. revised this manuscript. All authors have read and approved the final manuscript.

## CONFLICT OF INTEREST STATEMENT

The author Yanke Lin is an employee of Guangdong TCRCure Biopharma Technology Co., Ltd. but has no potential relevant financial or non‐financial interests to disclose. The other authors have no conflicts of interest to declare.

## ETHICS STATEMENT

The author declares that ethics approval was not needed for this study.

## Data Availability

All data are available upon request.
